# Duodenal damage complicating percutaneous access to kidney

**DOI:** 10.1590/S1516-31802000000400008

**Published:** 2000-07-07

**Authors:** Antonio Corrêa Lopes, Marcos Tobias-Machado, Roberto Vaz Juliano, Marco Aurélio Lipay, Milton Borrelli, Eric Roger Wroclawski

**Keywords:** Percutaneous Nephrostomy, Duodenal Fistulae, Duodenopathies, Nefrostomia percutânea, Duodenopatias, Fístula duodenal

## Abstract

**CONTEXT:**

Since the first percutaneous nephrostomy performed by Goodwin in 1954, technical advances in accessing the kidneys via percutaneous puncture have increased the use of this procedure and thus the complications too. Among these complications, digestive tract damage is not common.

**DESIGN:**

Case report.

**CASE REPORT:**

We report a duodenal lesion that was corrected using surgical exploration and we touch on the therapeutic options, which may be conservative or interventionist. We chose conservative treatment, which has been approached in diverse manners in the literature.

## RESUMO

**CONTEXTO:**

Desde a primeira nefrostomia por punção realizada por Goodwin em 1954, os avanços técnicos no acesso percutâneo ao rim aumentaram a freqüência destes procedimentos e, como conseqüência, maior número de complicações. Dentre essas, as lesões do trato digestivo são raras.

**TIPO DE ESTUDO:**

Relato de caso.

**RELATO DE CASO:**

Relatamos lesão duodenal que foi corrigida com exploração cirúrgica e abordamos as opções terapêuticas que podem ser conservadora ou intervencionista. Optando-se pelo tratamento conservador, encontra-se nas publicações diferentes formas de abordagem.

## INTRODUCTION

Percutaneous surgery of the kidney is the gold standard treatment for temporary urinary diversion in ureteral obstruction due to gynecological cancer.

Knowledge of possible complications of this surgery is very important. Duodenal damage is an uncommon complication described in the literature. The treatment of this condition has different approaches, ranging from the conservative (drainage, nasogastric tube and parenteral nutrition) to the repair of the lesion by laparotomy.

We describe a patient with duodenal damage due to an accident during kidney puncture, which was treated by suture of the lesion.

## CASE REPORT

The patient was a 68-year-old woman with stage IIB uterine cervical carcinoma who had undergone radiotherapy 1 year before and developed post-therapy renal failure. An unsuccessful attempt to pass a double J catheter was first made, followed by right percutaneous ultrasound-guided nephrostomy. There was good urine drainage for six hours, but then the catheter output ceased. An unsuccessful attempt to remove the obstruction was made, followed by a second ultrasound-guided puncture, which was difficult due to insufficient renal pelvis dilation. During the procedure, enteric fluid leaked through the Amplatz polyethylene dilator. Due to this intercurrence and to unsuccessful attempts to shunt the excretion pathway, laparotomy was performed in the lower back to permit nephrostomy and explore the peritoneal cavity.

Operation findings included clots in the excreting pathway, blood and serous fluid in the cavity, and a 0.5-cm lesion on the 2nd duodenal portion. Once the nephrostomy was performed, the duodenal wall was sutured on two plans, and two drains, one periduodenal and one perirenal, were kept. The patient remained fasting until the 5th postoperative day, when diet was reintroduced and the perirenal drain was removed. The periduodenal drain was removed on the 6th postoperative day, and the patient was discharged on the 7th post-operative day with a functioning nephrostoma. Thirty days later she had no pyelocalyx dilation.

## DISCUSSION

Recent reports on large patient series have shown percutaneous puncture of the excretion pathway to be a relatively safe procedure, with complication rates ranging from 5 to 8 percent. Involvement of organs adjacent to kidneys is not common, but is reported in isolated cases. Damage to the gastrointestinal (GI) tract is extremely rare, most often involving the colon.^1,2^ Fifteen cases of duodenal damage associated to percutaneous procedures have been described so far.^1-3^

Timing of diagnosis includes:

1. Early diagnosis by retroperitoneal collection with renoduodenal fistula (using contrast-enhanced imaging of the excretion pathway and GI tract).
2. Diagnosis immediately after the procedure (puncture of enteric fluid and visualization of enteric mucous membrane folds).

Surgery is the classic approach for trauma-associated duodenal lesions. Conservative management is reported in some cases using percutaneous intraduodenal catheterization with peripheral drainage, 10 to 14 days’ fasting, and parenteral feeding.^1-3^ Culkin also points out the fact that this condition is benign, usually being resolved via a nasogastric tube, nephrostoma, and 14 days’ fasting with no parenteral feeding.^4^

In our patient, access via laparotomy on the lower back allowed us to clear the renal pelvis, to adequately place the nephrostomy catheter, to explore and repair the duodenal lesion, and to clear the cavity, with relative benefit and shorter hospital stay. Access to the peritoneal cavity by lower back laparotomy enabled us to adequately explore all possibly damaged abdominal organs.

When access to the distal portion of the ureter is obtained, enabling ureteral catheterization or functional nephrostomy, and perioperative diagnosis of duodenal damage, conservative treatment by placing a duodenal tube for peripheral drainage of the lesion may be carried out with good results.
